# The Influence of the Enhanced Vector Meson Sector on the Properties of the Matter of Neutron Stars

**DOI:** 10.1371/journal.pone.0106368

**Published:** 2014-09-04

**Authors:** Ilona Bednarek, Ryszard Manka, Monika Pienkos

**Affiliations:** 1 Department of Astrophysics and Cosmology, Institute of Physics, University of Silesia, Katowice, Poland; 2 Independent Researcher, Katowice, Poland; University of Oxford, United Kingdom

## Abstract

This paper gives an overview of the model of a neutron star with non-zero strangeness constructed within the framework of the nonlinear realization of the chiral 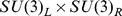 symmetry. The emphasis is put on the physical properties of the matter of a neutron star as well as on its internal structure. The obtained solution is particularly aimed at the problem of the construction of a theoretical model of a neutron star matter with hyperons that will give high value of the maximum mass.

## Introduction

Recent neutron star observations make it possible to distinguish in the entire population of neutron stars a class of massive stars. Particularly important data that indicate the existence of massive neutron stars concern the 3.1 ms radio pulsar in the binary system J1614-2230 [1] and the binary pulsar J0348+0432 [2] with the rotation period P = 39.1 ms. These binary systems possess features that make them extremely important for astrophysics. PSR J0348+0432 includes a neutron star and a low mass white dwarf companion (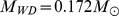), the orbital period of this system is 2.4 hr. The estimation of the neutron star mass based on radio timing and the precise spectroscopy of the white dwarf companion gives as a result 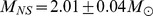. This binary system due to its special characteristics, namely: the neutron star and the low-mass white dwarf and tight, relativistic orbit is expected to provide information on the orbital decay due to the emission of gravitational waves. PSR J1614-2230 is a binary system that consists of a neutron star and a white dwarf. Measurements of the general relativistic time delay (Shapiro effect) allows for very accurate estimation of the mass of both components: neutron star mass 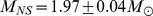 and white dwarf mass 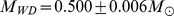.

In the light of these observations, it is important to know the evolutionary path that leads to the formation of a neutron star with such large mass. The most general scenario predicts that the formation of a cold catalysed object is preceded by a very specific phase of a hot neutron star with trapped neutrinos, which defines a proto-neutron star [3], [4]. Understanding the evolution of neutron stars requires a detailed analysis of the properties of a proto-neutron star matter, especially the heat transport [5].

The existence of massive neutron stars entails important consequences for the equation of state (EoS) of dense nuclear matter. In general, the precise measurements of pulsar masses put constraints on the form of the EoS and make problematic the existence of exotic particles such as hyperons and quarks [6] in the very inner part of a neutron star. Realistic models of neutron stars provide stratification of their internal structure by distinguishing two main components: a crust and a core. The crust, that splits into the outer and inner part, describes the outer layer of a neutron star with subsaturation densities and contains only a small percentage of a neutron star mass [7]. The description of the core of a neutron star is modelled on the basis of the EoS of dense nuclear matter in a neutron-rich environment [8] having a density that ranges from a few times the saturation density (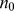) to about an order of a magnitude higher and at such densities hyperons are expected to emerge [9].

Despite the fact that neutron star matter is directly affected by the nature of strong interactions, it is not possible to give its description on the basis of quantum chromodynamics (QCD) even though it is fundamental theory of strong interactions. The description of nuclear matter is based on different models and with the use of different approaches. The commonly adopted method is the mean field approximation either relativistic or nonrelativistic. In the latter case the Skyrme forces [10], which contain parameters that have been established by adjusting nuclear matter and finite nuclei properties, yields very good results. In the microscopic approach the models of the nuclear matter EoS has been constructed with the use of the Brueckner-Hartree-Fock (BHF) approximation with the employed realistic two-nucleon interactions. In order to obtain the correct description of nuclear matter properties a phenomenological three-nucleon interactions was introduced [11], [12]. The paper [13] comprises a first attempt to describe a properties of strangeness-rich matter within Brueckner theory. The extension of the analysis for the 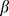-stable nuclear matter with hyperons was given in the paper [14]. Further development of the method that allows one to model strangeness-rich nuclear matter in this microscopic approach takes into account not only hyperon-nucleon (YN) but also hyperon-hyperon (YY) interactions [15]. In the context of massive neutron stars that contain hyperons the effect of the hyperonic three-body forces was estimated [16]. Calculations of the EoS performed on the basis of BHF approximation with additional phenomenological density-dependent contact terms do not result in a correspondingly large neutron star masses but form the basis for further more sophisticated analysis.

Observations of neutron stars with masses of the order of 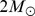 also do not excluded the existence of hybrid neutron stars with quarks nucleated in their inner cores. Efforts were done to analyse conditions under which the appearance of a quark core does not cause too strong softening of the EoS. There were several attempts to construct models of hybrid stars that satisfactorily reproduce the mass of a neutron star at the level of 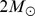. The differences between them result from the form of the EoSs that were used to describe the hadronic and the quark phases. Models that were used to describe the hadronic phase are the phenomenological models with the most popular relativistic mean field models [17] with different parameterizations and the microscopic Brueckner-Hartree-Fock approach [18]–[20]. The deconfined quark phase was modelled for example on the basis of the MIT bag model or the Nambu-Jona-Lasinio (NJL) model [20]–[22]. However, it was found that the transition to the quark matter phase is possible only for selected EoSs and parameterizations. Detailed analysis performed for the model of a hybrid star constructed on the basis of microscopic Brueckner-Hartree-Fock approach for hadronic phase and the MIT bag model, the NJL model and chromodielectric model for the quark phase are given in the following papers [18]–[20]. Also the effect of a hyperonic three-body force on the metastibility of compact stars was investigated leading to the estimations of the maximum mass of a hybrid star at the level of 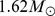 [20]. Results that were obtained for the hybrid star model with the quark matter phase described in the framework of a standard color superconducting NJL model and the hadronic phase constructed on the basis of the Dirac-Brueckner-Hartree-Fock EoS for the Bonn-A potential indicate the possible existence of massive hybrid stars [23].

The purpose of this paper is to study the impact of hyperons on the properties of the matter of neutron stars, and on their structure. In general, analysis of the role of strangeness in nuclear structure in the aspect of multi-strange system is of great importance for both nuclear physics and for astrophysics, and leads to a proper understanding of the properties of a hyperon star. The aim of this paper is an accurate analysis of the properties of neutron stars with particular regard to their internal structure. The key issue to be examined relates the determination of the factor which in the considered model is responsible for the stiffening of the EoS. Another aim of this work is to provide understanding on the impact of the model with the extended vector meson sector on the internal structure of a neutron star.

The relativistic approach to the description of nuclear matter developed by Walecka [24] is very successful in describing a variety of the ground state properties of finite nuclei. Although the original Walecka model properly describes the saturation point and the data for finite nuclei, it has been insufficient to properly describe the compression modulus of symmetric nuclear matter at saturation density. The nonlinear self-interactions of the scalar field (the cubic and quartic terms) were added in order to get an acceptable value of the compression modulus [25], [26]. Additionally the inclusion of a quartic vector self-interaction term softens the high density component of the EoS [27]. The estimation of the incompressibility coefficient of symmetric nuclear matter 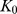, which is made on the basis of recent experimental data, points to the range 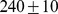 MeV [28].

Models that satisfactorily reproduce the saturation properties of symmetric nuclear matter lead to considerable differences in a case in which asymmetry dependence is included [29], [30]. Thus, the proper model of the matter of neutron stars requires taking the effect of neutron-proton asymmetry into consideration. This, in turn, leads to the inclusion of the isovector meson 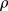. The standard version of the introduction of the 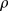 meson field is of a minimal type without any nonlinearities. This case has been further enlarged by the nonlinear mixed isoscalar-isovector couplings, which modify the density dependence of the 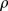 mean field and the symmetry energy [31]–[33]. The analysis of the nonlinear models should include results obtained for the relativistic FSUGold parametrisation [34].

A theoretical description of strangeness-rich nuclear matter requires the extension of the model to the full octet of baryons and additional meson fields were introduced to reproduce the hyperon–hyperon interaction. The model that is considered is constructed on the basis of the hadronic 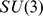 theory, which naturally includes nonlinear scalar and vector interaction terms. The characteristic feature of the model is the very special form of the vector meson sector, which permits more accurate description of asymmetric strangeness-rich neutron star matter [35]. The primary goal of this paper is to maximize the understanding of the influence of the nonlinear vector meson couplings on the form of the EoS and through this on a neutron star structure.

Having obtained the EoS the analysis of the maximum achievable neutron star mass for a given class of models can be performed. Observational results limit the value of a neutron star mass and thereby put constraints on the EoS of high density nuclear matter. Recent observations point to the existence of a high maximum neutron star mass [1], [2], what is inconsistent with theoretical models that involve hyperons.

### The model

Recent observations of the binary millisecond pulsars J1614-2230 [1] and J0348+0432 [2] have led to the precise estimation of neutron star masses: 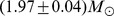 and 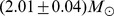. This places the maximum neutron star mass at rather high values and rules out most of the EoSs with hyperons as models that involve exotic particles predict maximum neutron star masses well below the stated values. There is a need to analyse whether it is possible to construct an EoS of neutron star matter that gives adequately high maximum mass despite including hyperons.

In this paper a description of nuclear matter based on an effective model constructed within the framework of the nonlinear realization of the chiral 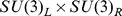 symmetry [36]–[38] is giving. Details can be found in the papers [35], [39]. Baryons and mesons constitute the basic degrees of freedom of the model, and consequently the Lagrange density function 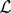 splits into parts that are adequate to describe baryon 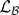 and meson 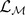 sectors supplemented by the term that represents baryon–meson interactions 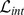, and takes the form 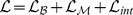. The meson sector of the considered model includes spin zero and spin one meson states. Nonets of different meson types, spin zero (scalar) and spin one (vector), can be written as the sum of the singlet and octet matrixes 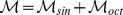. Under the assumption of SU(3) symmetry, a very general form of the interaction Lagrangian 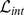 includes a mixture of the symmetric (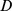 -type) and antysymmetric (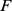 -type) couplings and the 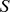 -type coupling that denotes the meson singlet state interaction
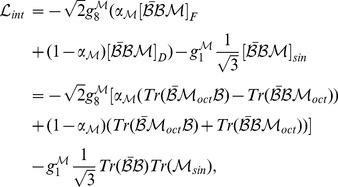
(1)


where the explicitly given baryon matrix 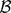 has the form
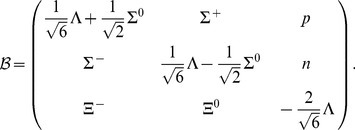
(2)


Generally, the baryon–meson interaction is characterised by the following coupling constants: the octet 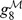 and singlet 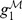 coupling constant, the parameter 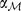, which stands for the 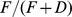 ratio, and a mixing angle 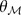 that relates the physical meson fields to the pure octet and singlet states. The index 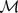 concerns the scalar (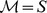) and vector (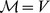) mesons (pseudoscalar and axial vector mesons, which have a vanishing expectation value at the mean field level, are not considered in this model). In the case of the scalar meson sector baryon masses are generated by the vacuum expectation value that is attained by two scalar meson fields and the parameters 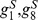, and 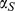 have been chosen to fit the experimental values of the baryon-octet masses [36], [37]. Considering the vector meson sector of this model the octet matrix
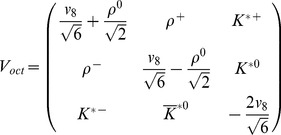
(3)


supplemented with the singlet state
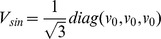



comprises the vector meson nonet. The combinations of the unphysical SU(3) singlet (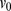) and octet (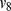) states produce the physical 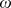 and 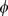 mesons
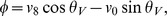


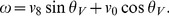
(4)


The 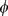 meson taken as pure 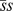 state leads to the ideal mixing with 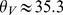
°. If the determination of the baryon–vector meson couplings bases on the assumption that nucleons do not couple to the 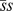 meson, then 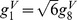. In the limit 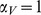 (only the 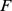 -type coupling remains), the coupling constants are related to the additive quark model and the vector meson coupling constants are given by the following relations:
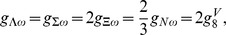


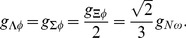
(5)


The high density limit of the EoS of dense matter in neutron star interiors is dominated by the contribution that come from the baryon number density, which justifies the construction of a model that includes a broad spectrum of mixed vector meson couplings. The extended vector meson sector, which stems from the SU(3) invariants, can be written in the following form
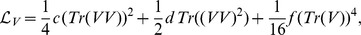



where 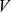 represents the matrix of the vector meson fields, and 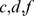 are the general coefficients that have been determined by assuming that in the case of a neutron star with zero strangeness, the model described by the TM1 parameter set [27] is recovered; 
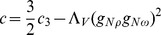
, 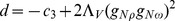, 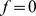. This assumption leads to the Lagrangian function, which embodies contributions from the baryon and meson sectors supplemented by the parts that describe the baryon and meson interactions
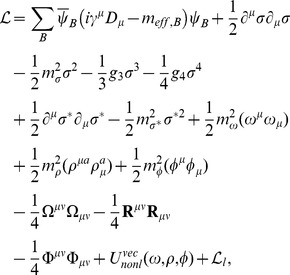



where the covariant derivative equals 
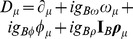
, 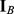 denotes isospin of baryon 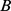. The baryon effective mass is defined as follows 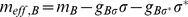, while 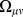, 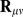, and 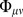 are the field tensors of the 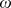, 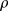, and 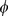 mesons. A proper description of hyperon–hyperon interaction requires the presence of hidden strangeness mesons: scalar (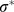) and vector (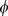). The Lagrangian function (6) describes the 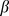-equilibrated neutron star matter; thus there is also a need to consider the Lagrangian of free leptons 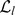


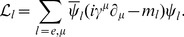
(7)


All nonlinear vector meson couplings that occur in this model have been brought together in the form of a vector potential
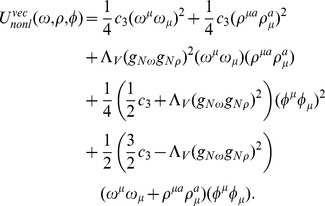



The description of dense, hyperon-rich nuclear matter given by the Lagrangian (6) in the case of non-strange matter is reduced to the standard TM1 model with an extended isovector sector. This extension refers to the presence of the 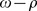 meson coupling and enables modification of the high density limit of the symmetry energy. The strength of this coupling is characterised by parameter 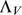. For each value of parameter 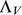, the parameter 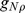 has to be adjusted to reproduce the symmetry energy 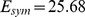 MeV at 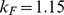 fm^−1^ [40].

### The equation of state

The mean field approach has been adopted to calculate the EoS. In this approximation, meson fields are separated into classical mean field values: 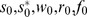 and quantum fluctuations, which are neglected in the ground state:
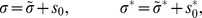


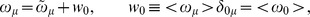


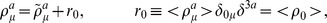


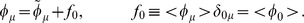



The preferable attribute of the considered model is its very diverse vector meson sector, which allows one to study the relevance of different vector meson couplings for the form of the EoS. The Lagrangian function (6) makes it possible to calculate the equations of motion from the corresponding Euler-Lagrange equations. The obtained results, written in the mean field approximation, take the form:
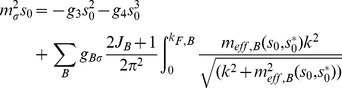
(9)

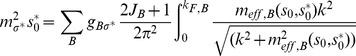
(10)

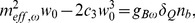
(11)

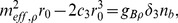
(12)

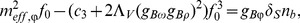
(13)

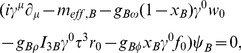
(14)


where 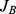 and 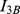 denote the spin and isospin projection of baryon 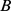, 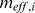, (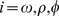) are effective masses assigned to vector meson fields:
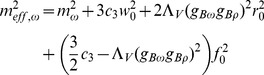
(15)

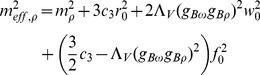
(16)

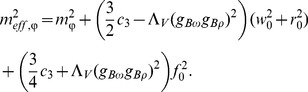
(17)


Analysis of the equations of motion raises the issue of the medium effects on the properties of the hadronic matter namely the effective baryon and vector meson masses. Considering the case in which nucleon mass depends only on non-strange condensate and referring to the Walecka model, the relation for the reduced effective baryon masses in the medium in the relativistic mean field description [31] can be formulated as
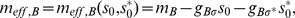
(18)


where the terms 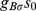 and 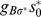 represent the modification of baryon masses due to the medium and 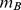 denotes baryon masses generated by the vacuum expectation values attained by scalar meson fields [36], [37].

The source terms in equations (11–13) can be expressed by introducing the parameters 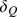 and 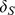, which measure the contributions of strange (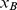) and non-strange quarks:
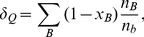
(19)

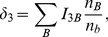
(20)

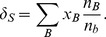
(21)


Thus, the number density of 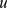 and 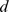 quarks refers to 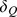, whereas the number density of strange quarks is given in terms of the parameter 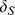. The parameter 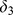 aims to evaluate the difference between the density of 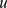 and 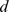 quarks, 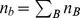 denotes the total baryon number density. The baryon–vector meson coupling constants determined from the symmetry relations have been summarised in Table 1. The parameters 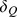, 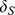 and 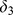 will be further used to define the asymmetry parameter 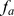 and strangeness content 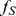 of the matter of a neutron star:

**Table 1:** Vector meson coupling constants.

Baryon (B)	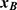	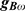	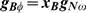	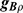
n	0	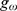	0	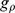
p	0	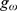	0	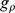
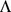	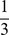	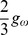	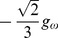	0
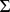	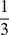	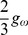	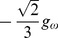	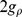
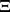	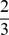	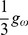	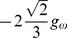	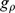

Baryon–vector meson coupling constants, 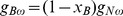 and 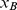 counts the contribution of strange quarks, 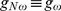 and 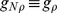.



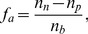
(22)

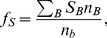
(23)


where 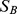 denotes the strangeness of baryon 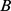 (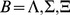). The numerical solution of the equations of motion, which depends on the form of the effective vector potential 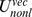, has been limited to the result with only one single real solution. The demand for the existence of one real solution puts constraints on the value of parameter 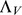. Such an analysis in the simpler case of symmetric nuclear matter leads to an equation that relates 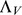 and 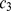 parameters and enables the critical value of parameter 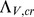 to be estimated
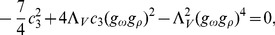
(24)


where 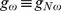 and 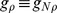.

The existence of one single real solution is not satisfied for 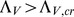. The critical value of the parameter 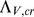 calculated for the selected parameterisations are collected in Table 2. In order to calculate the energy density and pressure of the nuclear matter, the energy momentum tensor 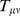, which is given by the relation

**Table 2:** The limiting values of the parameter 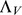.

Parameter set	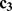	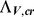	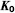 (MeV)
NL3 [61]	0	−	271
FSUGold [34]	418.39	0.0517	230
TMA [62]	151.59	0.0318	318
TM1 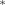 [63]	134.624	0.0215	281.1
TM1 [27]	71.3	0.0156	281.1
TM2 [64]	84.5318	0.0186	343.8

The limiting value of the parameters 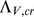 and 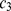 together with the incompressibility of the symmetric nuclear matter 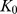 taken at the saturation density calculated for the chosen parameter sets.



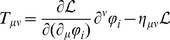
(25)has to be used. In equation (25) 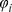 denotes the boson and fermion fields. The energy density 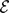 is equal to 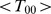, whereas the pressure 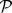 is related to the statistical average of the trace of the spatial component 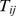 of the energy momentum tensor. Calculations done for the considered model lead to the following explicit formulas for the energy density and pressure:
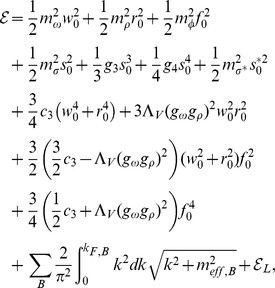


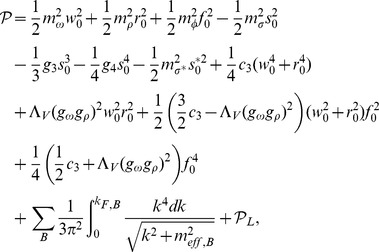
(27)


where 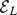 and 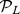 denote the contributions coming from leptons.

### Coupling constants

Understanding the nature of interactions between baryons is a decisive factor for the properties of neutron stars especially in the case when hyperons are included. A precise description of baryon interactions in the strange sector of the model is essential for the correct construction of the EoS. However, the incompleteness of the experimental data intensifies the uncertainties that are connected with the evaluation of coupling constants that involve strange baryons. In general, there are very few data available to describe hyperon–nucleon (YN) and hyperon–hyperon (YY) interactions.

Hyperon–vector meson coupling constants are taken from the quark model. They are summarised in Table 1. In the scalar sector, the scalar couplings 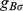 of the 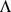, 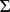 and 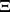 hyperons require constraining in order to reproduce the estimated values of the potentials felt by a single 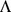, 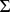 and 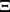 in the saturated nuclear matter. In the case of 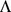 hypernuclei there is a considerable amount of data on binding energies and single particle levels allowing the identification of the potential felt by a single 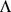 in nuclear matter in the range 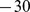 MeV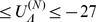 MeV [41], [42]. Considering the 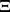 hypernuclei current knowledge about the interaction of 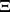 hyperons with nuclei is very limited. Dover and Gal [43] based on early emulsion data indicated an attractive 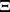 -nucleus potential in the range 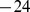 MeV 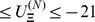 MeV. This result agrees with theoretical predictions for 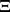 in nuclear matter obtained in the model D of the Nijmegen group [44]. However, the missing-mass spectra of a double-charge exchange reaction 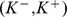 on a 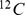 target have suggested the 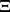 well depth of 14-16 MeV (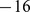 MeV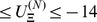 MeV) [45], [46]. Data related to the 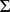 -nucleon interaction are controversial. Analysis of the experimental data indicate a repulsive 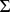 -nucleus potential [47], with a substantial isospin dependence [48]. In the case of YY interactions, the only sources of information are the double-strange hypernuclear systems. Several events have been identified that suggest an attractive 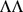 interaction. The most promising results, known as the NAGARA event [49] with the 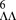
He hypernucleus, indicate that the 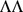 interaction is weakly attractive. The determined value of this potential at the level of 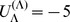 MeV permits a parameter set which reproduces this weaker 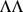 interaction to be estimated [39].

The potential that describe hyperon–nucleon and hyperon–hyperon interaction can be written in the form that involves both the scalar and vector coupling constants
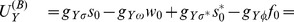


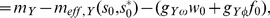
(28)


where 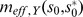 is the effective mass and 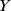 stands for the 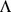, 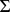 and 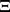 hyperons. For the determination of the 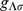, 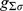 and 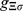 coupling constants, the following values of the potentials were used
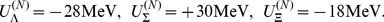
(29)


In general the coupling constants 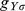 can be decomposed into two parts 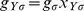, where 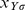 depends on the value of potential 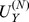. The coupling of hyperons to the strange meson 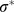 were obtained from the following relations [50]

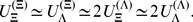
(30)


The scalar coupling constants are collected in Table 3.

**Table 3:** Scalar meson coupling constants.

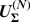 (MeV)	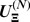 (MeV)	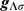	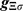	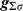	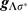	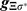	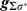
+30	−14	6.169	3.084	4.476	5.626	11.474	5.626
+30	−18	6.169	3.201	4.476	5.482	11.372	5.482
+20	−18	6.169	3.201	4.768	5.482	11.372	5.482
+10	−18	6.169	3.201	5.060	5.482	11.372	5.482
−10	−18	6.169	3.201	5.644	5.482	11.372	5.482
−20	−18	6.169	3.201	5.935	5.482	11.372	5.482
−30	−18	6.169	3.201	6.227	5.482	11.372	5.482

Scalar meson coupling constants 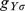 and 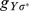 (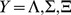) calculated for chosen values of the potentials 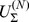 and 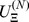.

### The symmetry energy

The TM1 parameter set (Table 4) that successfully describes the ground state properties of both finite nuclei and infinite nuclear matter was supplemented with the mixed nonlinear isoscalar–isovector 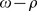 meson coupling, which provides the additional possibility of modifying the high density components of the symmetry energy. The remaining nuclear matter ground state properties were left unchanged. The symmetry energy is given by the relation
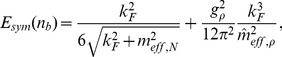
(31)


**Table 4:** The standard TM1 parameter set [27].

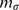 = 511.2 MeV	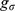 = 10.029	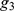 = 7.2325 fm 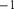
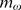 = 783 MeV	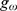 = 12.614	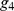 = 0.6183
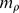 = 770 MeV	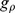 = 9.264	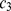 = 71.0375

where 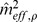 denotes the effective mass of the 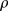 meson in the case of non-strange, symmetric nuclear matter.

The strength of the 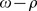 coupling is set by the 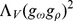 term and requires the adjustment of the 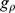 coupling constant to keep the empirical value of the symmetry energy 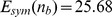 MeV at the baryon density 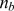, which corresponds to 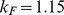 fm^−1^ [40]. The parameters 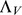 together with the adjusted value of the parameter 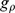 are presented in Table 5.

**Table 5:** TM1 parameter set - isovector sector.

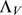	0	0.014	0.015	0.016	0.0165	0.017
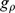	9.264	9.872	9.937	10.003	10.037	10.071
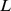 (MeV)	108.58	77.52	75.81	74.16	73.36	72.56

TM1 parameter set with the extended isovector sector. The extension includes the coupling between the 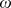 and 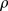 mesons: 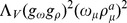. For each value of the parameter 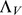 the 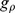 coupling constant has been adjusted to reproduce the empirical value of the symmetry energy 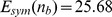 MeV at the baryon density 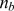 which corresponds to 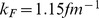 [40].

The density dependence of the symmetry energy can be expressed by coefficients that define the slope 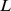 and the curvature 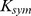 of the symmetry energy
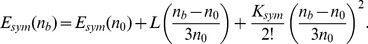
(32)


Equation (32) is a typical low density expansion, higher-order terms must be taken into account at suprasaturation densities. The performed calculations that were focused mainly on the slope parameter of the symmetry energy
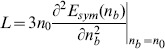
(33)


gave results that agreed with the experimental data (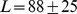 MeV [51]). These results are collected in Table 5. However, current estimation of the symmetry energy parameter based on theoretical, experimental and observational results narrows the range of 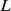 to 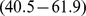 MeV [52].

## Results

**Figure 1:**
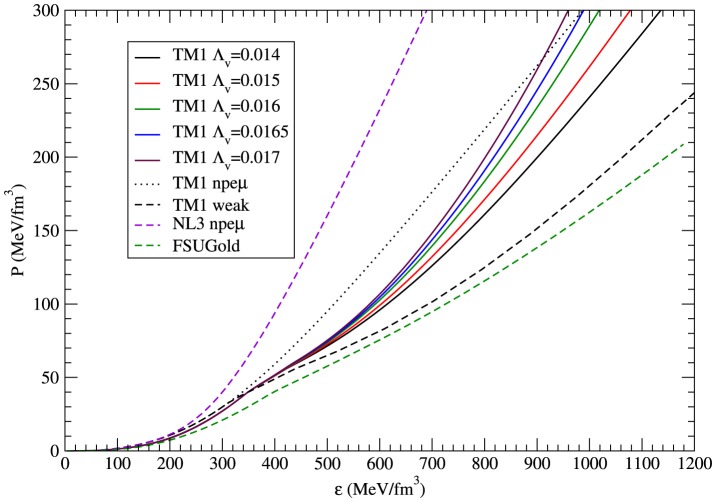
The pressure vs density calculated for selected parameterisations. The stiffest EoSs have been obtained in the case of non-strange matter for NL3 and TM1 parameter sets. The EoSs calculated for the extended nonlinear TM1 model for different values of the parameter 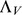 form a distinct class with the stiffest EoS obtained for 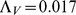. The softest EoS for strangeness rich neutron star matter represents the case of the standard TM1 model extended by the inclusion of strange mesons which have been introduced in a minimal fashion (TM1-weak model). The EoSs calculated for the extended nonlinear model for different values of parameter 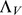 are located between these two curves. Nonlinear vector meson couplings stiffen the EoS which results in a higher value of the maximum mass.

The energy density and pressure given by relations (26) and (27) define the EoS. Numerical calculations, which were made for the TM1 parameterisation for both nonstrange and strangeness-rich matter, led to the solutions that are shown in Fig. 1. For this parameterisation a class of EoSs was obtained. Individual EoS is parametrized by the coupling constant 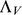, which determines the strength of the mixed vector meson interactions. The form of the EoSs allows one to compare the differences between various models.

An analysis performed for the nonlinear model with parameter 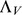, which ranges between 0.014 to 0.017, showed that the increase of 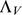 produces a stiffer EoS. For comparison, the EoSs for non-strange matter for NL3, TM1 and FSUGold parameterisations have been included. Thus, the stiffness of the EoS depends on the existence and strength of the mixed vector meson interactions, and the nonlinear model described by the Lagrangian function (6) makes it possible to construct a much stiffer EoS than the one obtained for the TM1-weak model (the abbreviation TM1-weak denotes the standard TM1 model extended to the full octet of baryons with two additional meson fields, which were introduced in a minimal fashion, to reproduce the hyperon–hyperon interaction).

**Figure 2:**
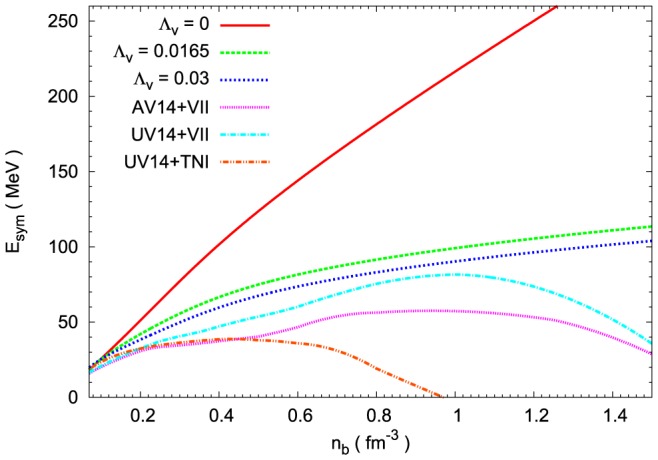
The density dependence of the symmetry energy. The density dependence of symmetry energy calculated for TM1 parameter set [27] for different values of the parameter 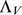. For comparison the results obtained for the AV14+VII, UV14+VII and UV14+TNI models [53] are included. The inclusion of 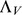 parameter softens the symmetry energy and its density dependence resembles that obtained for the realistic nuclear models.

In the case of nuclear matter an extended isovector sector comprises the 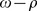 meson interaction and the parameter 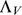 sets the strength of the 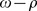 coupling. This term altered the density dependence of the symmetry energy. The standard TM1 parameterisation without 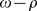 coupling gives as a result very stiff form of the symmetry energy. The inclusion of the 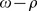 coupling softens the symmetry energy. The solutions were presented in Fig. 2. Calculations were done for rather high values of 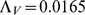 and 0.03. The interaction between 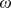 and 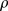 mesons leads to the solution, which approaches that obtained for the AV14 and UV14 models with the Urbana VII (UV VII) three nucleon potential. For comparison the form of the symmetry energy calculated for the UV14 plus TNI model was included [53].

**Figure 3:**
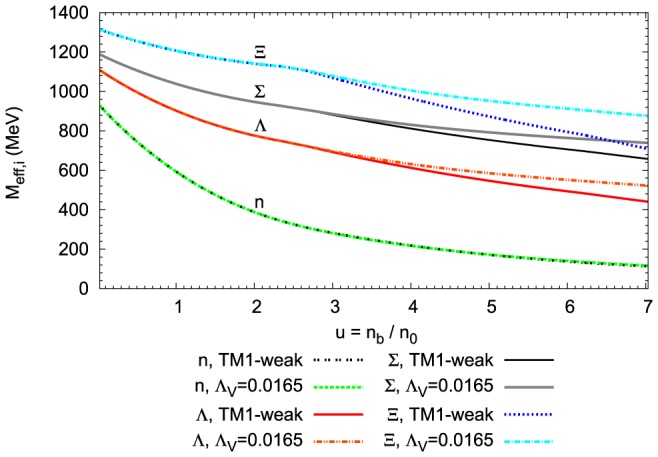
Medium modification of baryon masses. The effective baryon masses as a function of relative baryon density calculated for the extended nonlinear model, for 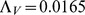 and for the TM1-weak model. The extended nonlinear model, in comparison to the TM1-weak one, leads to the higher effective strange baryon masses.

Interacting baryons are the basic components of the matter of neutron stars. The modification of baryon masses that arises from baryon interactions with the background nuclear matter is shown in Fig. 3. The numerical solutions predicted by equation (18) for the fixed value of parameter 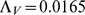 for both the nonlinear model and for the TM1-weak model, show reduced effective baryon masses.

**Figure 4:**
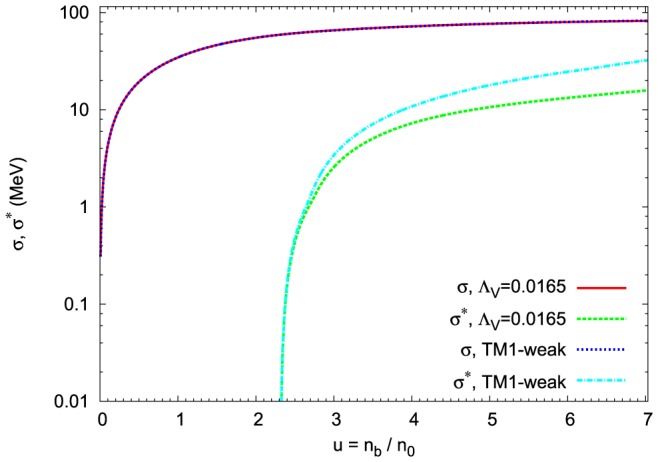
Density dependence of the scalar meson fields. The scalar mesons 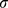 and 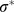 as a function of baryon density. For comparison the results obtained for the TM1-weak model have been included. There is a significant difference between the density dependence of the strange scalar meson for the TM1-weak and extended nonlinear models. The density dependence of scalar meson 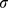 is left unchanged for both models.

The reduction of the nucleon mass in the nonlinear model is essentially the same as that obtained in the TM1-weak one. Thus, the influence of the nonlinear vector meson couplings on the nucleon effective mass is negligible. The effective masses of strange baryons in the case of the nonlinear model drop less rapidly than the effective masses obtained in the TM1-weak model. The behaviour of the baryon effective masses is governed by the density dependence of the scalar mean fields, which is presented in Fig. 4. The presence of nonlinear couplings between vector mesons modifies the density dependence of the strange scalar meson leaving the nonstrange scalar meson almost unchanged.

**Figure 5:**
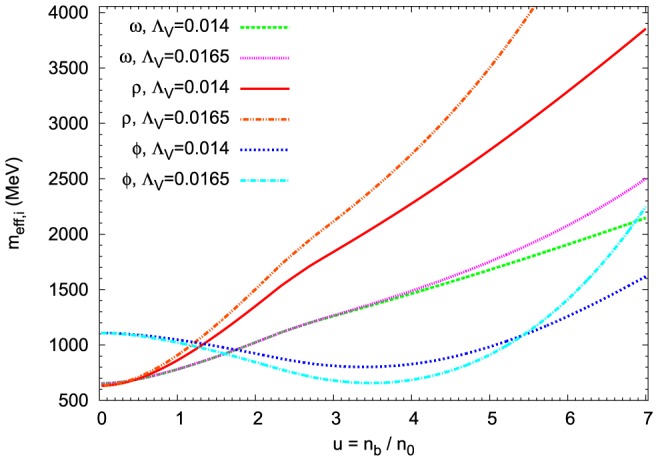
Medium modification of vector meson masses. The effective vector meson masses as a function of baryon density calculated for the extended nonlinear model. The results for 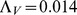 and 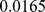 have been compared. In the case of 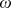 and 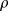 mesons the increase of 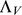 parameter results in their higher effective masses. While for the effective mass of the 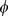 meson there exists a range of density where its effective mass decreases.

The in-medium reduction of baryon masses is equivalent to the modification of vector meson masses in the meson sector (Fig. 5). An analysis of the density dependence of the effective 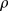 and 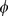 vector meson masses led to the conclusion that their modification was produced by a strong 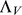 dependence, especially in the high density limit. The effective mass of the 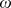 meson is almost independent of the value of parameter 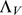.

**Figure 6:**
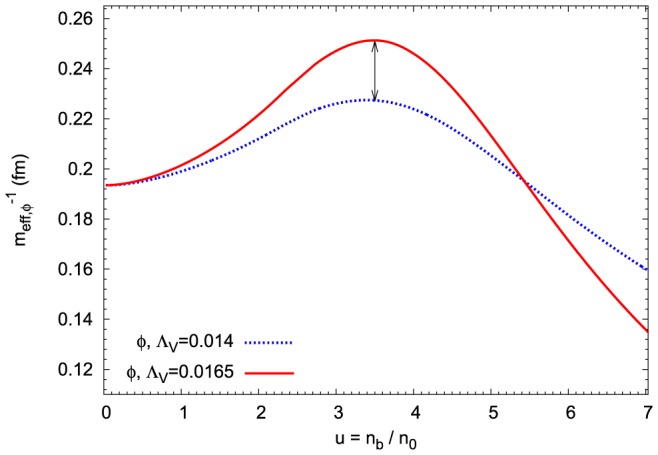
The range of hyperon-hyperon interaction. The density dependence of the factor 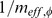 calculated for the nonlinear model, for 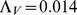 and 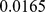. The behaviour of this parameter is correlated with the density dependence of 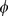 meson effective mass.

The stiffness of the EoS is characterised by the incompressibility of nuclear matter. In general, incompressibility comprises terms resulting from the kinetic pressure of Fermi gas and from the potential of the model. Analysing the strange sector of the model one can compare the factor that determines the difference between the strength of the effective repulsive and attractive forces. The scalar meson 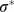 has been introduced in a minimal fashion thus 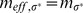. The strength of the effective repulsive force between strange baryons is mainly altered by the factor 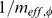. The influence of the 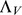 parameter on the density dependence of 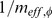 is depicted in Fig. 6. The increase of the parameter 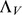 considerably enhances the strength of the repulsive force in the system.

The properties of asymmetric strangeness-rich matter of neutron stars are characterized by the parameters that define the strangeness content of the system 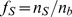, where 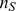 is the strangeness density and the isospin asymmetry 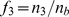, where 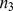 is the isospin density given by the relation
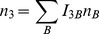
(34)


**Figure 7:**
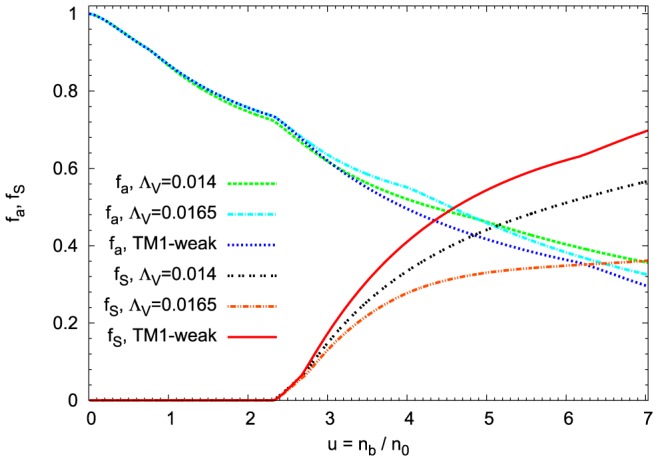
The isospin asymmetry and strangeness content of the matter of neutron stars. The asymmetry parameter 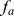 and the strangeness content of the system 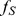 as a function of baryon density calculated for the extended nonlinear model for different values of the parameter 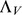. For comparison the result obtained and for the TM1-weak model has been included. In the case of the nonlinear extended model the matter of the neutron star is less symmetric and with the reduced strangeness content.

The density dependence of the asymmetry parameter 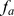 (22) and the strangeness content of the system 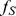 (23) are depicted in Fig. 7. The nonlinear model leads to a system with an enhanced asymmetry and a considerably reduced strangeness. An increase of parameter 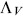 causes the matter to become more asymmetric. Charge neutrality and the condition of 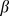 equilibrium (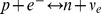) impose constraints on a neutron star composition. Assuming that neutrinos are not considered, since their mean free path is longer then the star radius, the following relation can be obtained: 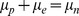.

Additional hadronic states are produced in neutron star interiors at sufficiently high densities when hyperon in-medium energy equals their chemical potential. The higher the density the more various hadronic species are expected to populate. In general weak reactions for baryons and the corresponding equations for chemical potentials can be written in the form
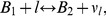
(35)

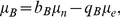
(36)


where 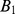 and 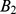 denote baryons, 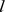 and 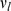 lepton and neutrino of the same flavor, whereas 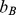 and 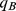 refer to baryon 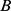 with baryon number 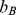 and charge 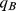. The above equations create relations between chemical potentials of particular hyperons
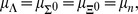


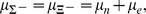


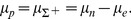
(37)


**Figure 8:**
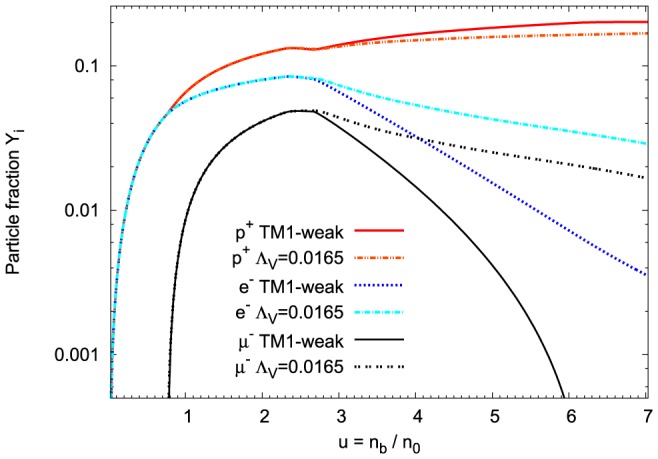
Nucleon and lepton concentrations in the matter of neutron stars. Relative concentrations of nucleons and leptons calculated for the extended nonlinear model, for the fixed value of the parameter 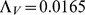. For comparison the results obtained for the TM1-weak model have been included. In the case of the TM1-weak model the lepton concentrations are reduced whereas the differences in neutron and proton concentrations are rather small.

**Figure 9:**
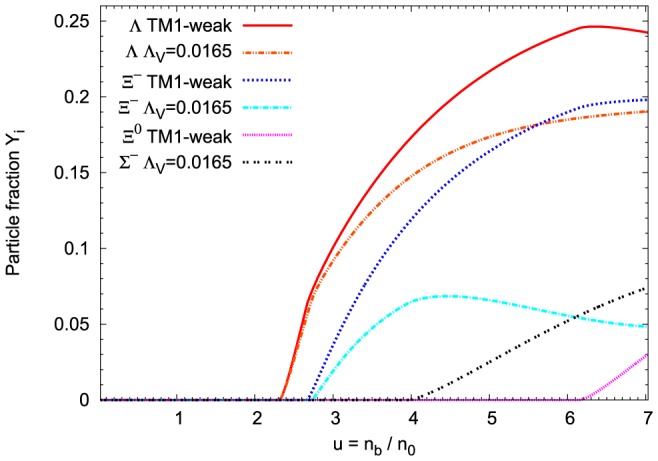
Concentrations of strange baryons in the matter of neutron stars. Relative concentrations of strange baryons calculated for the extended nonlinear model, for the fixed value of parameter 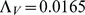. For comparison the results obtained for the TM1-weak model have been included. The extended nonlinear model leads to the reduced number density of 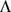 and 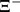 hyperons. Characteristic feature of this model is the appearance of 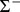 hyperons.

The presented equilibrium conditions determine all constituents of the matter of neutron stars. The concentrations of a particular component 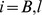 of the matter of a neutron star can be defined as 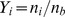, where 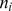 denotes the density of the component 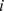 and 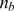 is the total baryon number density. The density fraction of nucleons and leptons as a function of the baryon number density for the fixed value of parameter 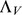 is presented in Fig. 8. The important findings concern the concentration of leptons, which are more highly populated in the case of the nonlinear model. Fig. 9 shows how the modification of the vector meson sector alters concentrations of strange baryons. The first hyperon that appears is 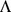 and it is followed by 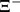 and 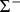. The appearance of negatively charged hyperons reduce the concentrations of leptons. This stems from the charge neutrality condition. However, the initial rapid increase in population of 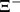 hyperons has been suppressed leading to significantly reduced concentration of 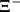 hyperons at sufficiently high density. For comparison the results obtained for the TM1-weak model have been included. It is evident that the additional nonlinear couplings between vector mesons modify chemical composition of the neutron star shifting the hyperon onset points to higher densities and reduces the strangeness content of the system.

**Figure 10:**
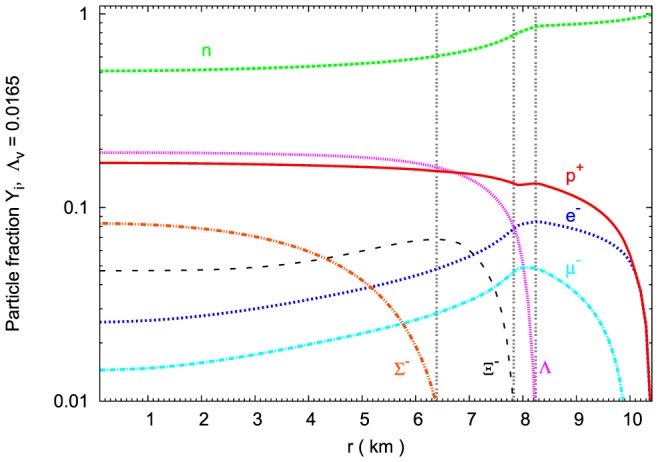
Chemical composition of the maximum mass configuration. The particle fraction 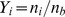 as a function of the radius of the neutron star, for the maximum mass configuration. Calculations have been done on the basis of the extended nonlinear model with 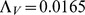. The dotted vertical lines point the threshold densities for particular hyperons.

A composition and concentrations of hyperons calculated in the nonlinear model can be traced in a chosen configuration of a neutron star. The composition of the core of the maximum mass configuration is depicted in Fig. 10. The number density of 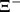 hyperons is reduced. However, an interesting feature of this model is the abundance of 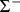 hyperons in the very inner part of the neutron star inner core.

The most essential feature of the model is connected with the fact that even in the presence of hyperons the obtained EoS is very stiff. Global neutron star parameters such as the mass and radius and the structure of a neutron star can be determined by the equation of hydrostatic equilibrium - the Tolman-Oppenheimer-Volkoff (TOV) equation:
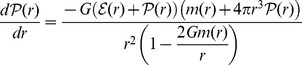


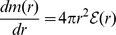


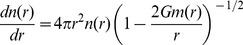
(38)


where 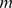 and 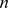 denote the enclosed gravitational mass and baryon number, respectively, 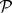 is the pressure and 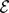 is the total energy density. In order to get the numerical solution of equation (38), the EoS has to be specified. A correct model of a neutron star is based on the assumption that its internal structure is composed of separate parts, thus the construction of the mass–radius relation requires taking into account additional EoSs that describe the matter of the inner and outer crust. For the outer and inner crusts the EoSs of Baym, Pethick, and Sutherland (BPS) [54] and Baym, Bethe and Pethick (BBP) [55] have been used respectively.

**Figure 11:**
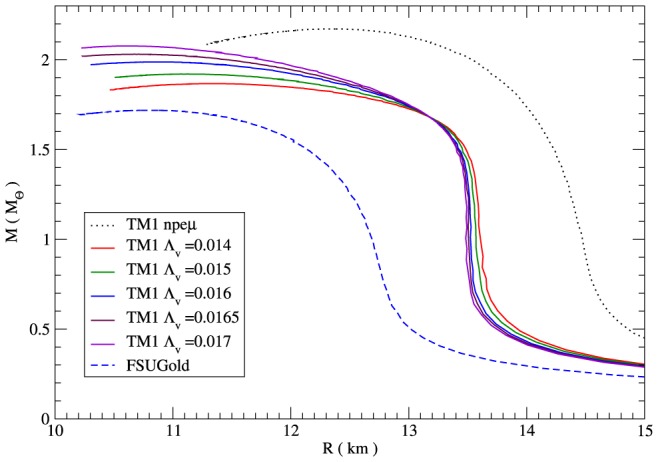
The mass-radius relations. The mass–radius relations calculated for the nonlinear model for different values of parameter 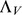. The results have been obtained for both the TM1 parameterisation in the case when the matter of the neutron star comprises only nucleons and for the FSUGold parameter set.

The results obtained for the set of EoSs calculated in this paper led to the mass-radius relations and permitted the value of the maximum mass to be determined which in a sense can give a measure of the impact of particular nonlinear couplings between vector mesons. The mass-radius relations obtained for varying values of parameter 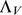 are shown in Fig. 11. The higher the value of 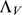, the higher the maximum mass.

### The influence of the 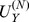 potentials

There are still significant uncertainties associated with the experimental data on the hyperon-nucleus interactions. Thus it is reasonable to investigate the effect of the hyperon-nucleus potential 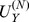 on the obtained results [56]. Particular attention was paid to the dependence of the EoS on the 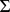 -nucleus potential 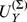.

**Figure 12:**
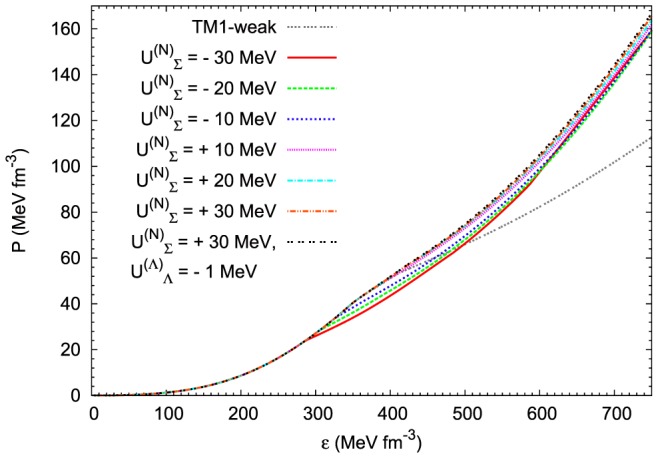
The influence of the 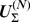 potential on the EoS. The pressure vs density calculated for different values of the 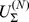 potential (attractive and repulsive), the case of a very weak 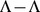 interaction (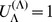 MeV) has been also included. For comparison the EoS calculated for the TM1-weak model has been shown. In the case of the extended nonlinear model the higher value of the repulsive 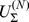 potential leads to the stiffer EoS.

Detailed calculations were done for the selected values of the 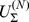 potential, assuming its both attractive and repulsive character. Calculations performed for the extended nonlinear model were resulted in a sequence of EoSs (Fig. 12). The stiffest one was obtained for the repulsive potential (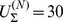 MeV). For comparison the EoS obtained for TM1-weak model with the repulsive 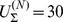 MeV potential was included. In order to study the influence of the 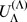 potential a model with an exaggerated value of the potential 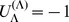 MeV was examined. The resulting change in the EoS is negligible. A similar conclusion can be drawn by examining the changes caused by the reduction in the potential 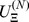. Calculations were done for two chosen values of the 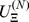 potential: 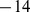 MeV and 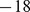 MeV.

**Figure 13:**
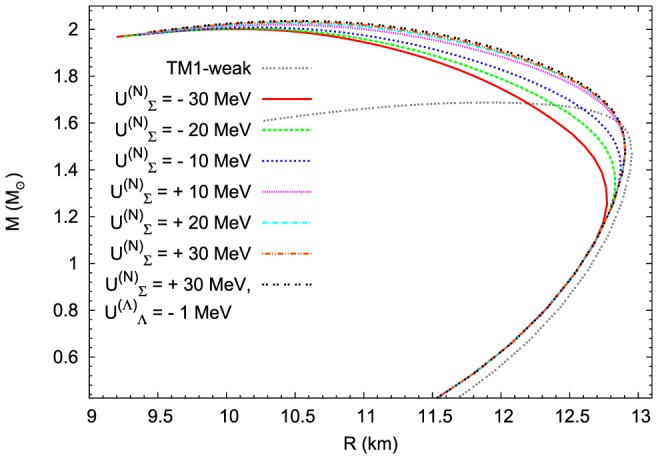
The influence of the 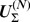 potential on the mass–radius relation. The mass – radius relations calculated for EoSs presented in Fig.12. In the case of the extended nonlinear model the higher value of the repulsive 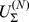 potential leads to the higher value of the neutron star maximum mass.

Taking into account the value of the maximum mass obtained for a given EoS the presented results support the conclusion that the value of the potential is not a factor that decisively influences the form of the EoS. A similar conclusion can be obtained by analysing the mass-radius relation (Fig. 13). The most promising results were obtained for the repulsive 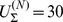 MeV potential which within the considered model leads to the highest value of the maximum mass.

**Figure 14:**
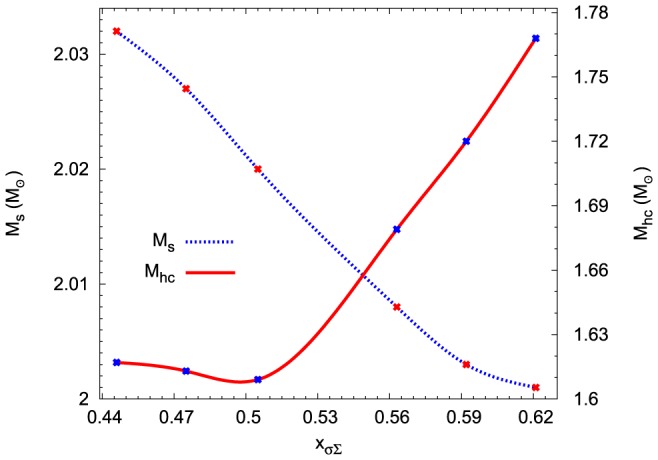
The influence of the 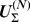 potential on the internal structure of the maximum mass configuration. The dotted line shows the dependence of the value of the maximum mass 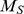 obtained for the given EoS on the parameter 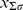. The parameter 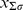 carries information about the hyperon-nucleon interaction. Solid line depicts similar relations for the mass of the hyperon core 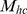.

**Figure 15:**
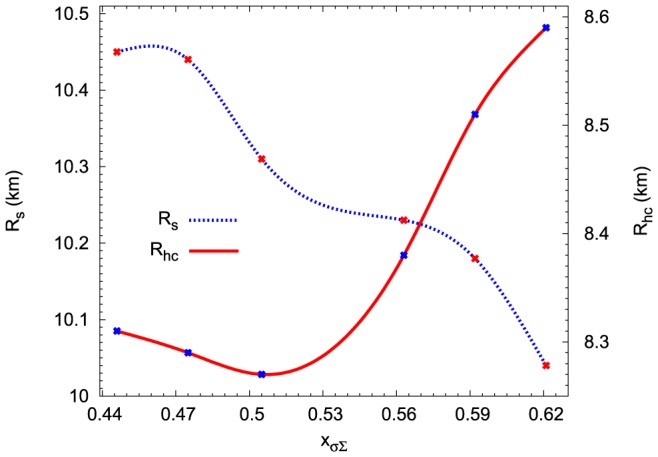
Neutron star and their hyperon core radii as functions of the hyperon-nucleon interaction. The dotted line shows the dependence of the radius of the maximum mass configurations 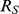 obtained for the given EoS on the value of parameter 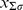. The solid line depicts similar relations for the radii of the hyperon core 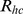.

Hyperons are distributed in the very inner part of a neutron star core therefore a schematic cross-section shows a hyperon core inside of a strangeness-rich neutron star. Information concerning properties of the internal structure of a neutron star are summarised in Fig. 14 and 15. These figures depict the dependence of both neutron stars and their hyperon cores on the 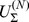 potential and strictly speaking on the value of 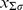 parameter and offer indications for interpreting the results of numerical calculations. Calculations were done for maximum mass configurations. The mass of the star increases when 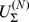 potential becomes more repulsive whereas the mass of the hyperon core decreases reaching a minimum value for the parameter 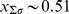 (Fig. 14). Similar behaviour has the radius of the maximum mass configuration. Results are presented in Fig. 15.

**Figure 16:**
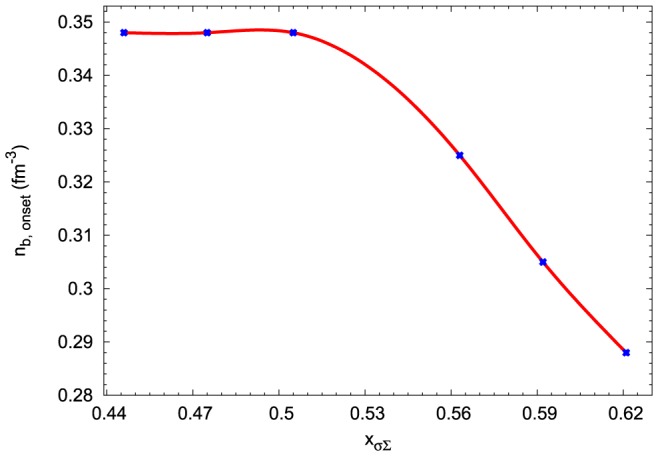
Hyperon onset points for the maximum mass configurations. The dependence of the hyperon onset points on the value of the parameter 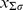.

**Figure 17:**
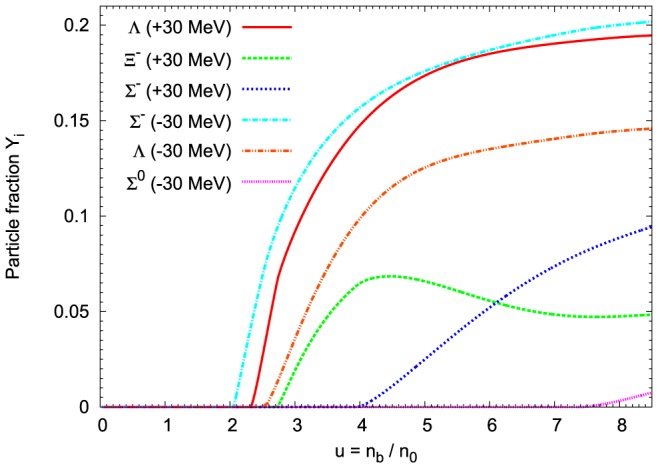
Hyperon concentrations for opposite signs of the 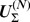 potential. Comparison of the relative concentrations of hyperons calculated for the extended nonlinear model for the repulsive and attractive 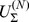 potential. In the case of the repulsive potential the first hyperon that appears is 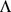 and it is followed by 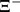 and 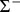, for the attractive potential hyperons appear in the following order: 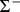, 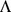 and 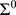.

One of the most important difference between the model constructed for the attractive 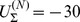 MeV and the repulsive 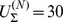 MeV potential is related to the chemical composition of the neutron star matter. The hyperon onset points depend on the character of the 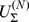 potential. Calculations performed on the basis of the extended nonlinear model indicate that the enhancement of the attractive 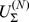 potential shifts the hyperon onset points to lower densities, whereas the change of the repulsive potential leaves the hyperon onset points almost unchanged (Fig. 16). In the case of attractive potential the first hyperons that appear in neutron star matter are 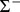 hyperons and they are followed by 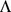 hyperons. Such a scheme of the emergence of hyperons is changed in the nonlinear model with the repulsive 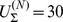 MeV potential. The first hyperons that appear are 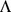 hyperons and successively 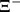 and 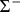 hyperons. The relative concentrations of hyperons calculated for both attractive and repulsive 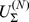 potential are presented in Fig. 17. The results of numerical calculations obtained for different values of the potential 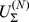 were compiled in Table 6. These results for a given EoS include the value of the maximum mass, the radius of the maximum mass configuration, the radius and mass of the hyperon core and the onset point of hyperons. For comparison the model with the 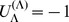 MeV was included.

**Table 6:** The influence of the 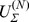 potential on neutron star parameters.

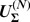 ( 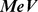 )	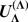 ( 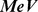 )	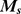 ( 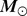 )	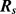 ( 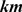 )	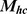 ( 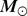 )	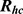 ( 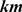 )	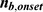 ( 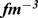 )
+30	−1	2.036	10.47	1.620	8.32	0.348
+30	−5	2.032	10.45	1.617	8.31	0.348
+20	−5	2.027	10.44	1.613	8.29	0.348
+10	−5	2.020	10.31	1.609	8.27	0.348
−10	−5	2.008	10.23	1.679	8.38	0.325
−20	−5	2.003	10.18	1.720	8.51	0.305
−30	−5	2.001	10.04	1.768	8.59	0.288

Neutron star maximum masses and corresponding radii calculated for the chosen values of the potentials 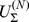 and 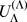. 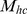 and 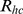 denote the mass and radius of a hyperon core for a given maximum mass configuration, 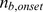 denotes the baryon density at which hyperons appear in the system (hyperon onset point).

The problem connected with the impact of the 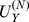 and 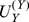 potentials on the gross parameters of a hyperon-rich neutron star has been developed so far. Solution of the TOV equations (38) can also provide information on the influence of the hyperon-nucleon and hyperon-hyperon interactions on the internal structure of a given star. The purpose of such an analysis is to find similarities and differences in the internal structures of neutron stars whose models were obtained for significantly different equations of state. Comparison of the internal structure of stars was carried out for the configuration of the maximum mass achievable for the given model, for the cases with different values of the 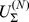 potential.

**Figure 18:**
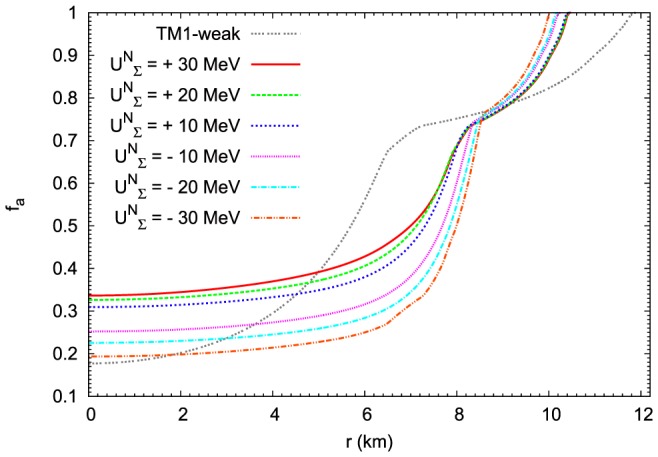
The profiles of the asymmetry of the matter of neutron stars. The asymmetry of the matter of neutron stars for the maximum mass configurations. Calculations have been done for the extended nonlinear model for the fixed value of the parameter 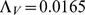. Profiles of the asymmetry parameter 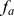 have been estimated for the 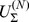 potential ranging from 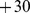 MeV to 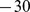 MeV. The asymmetry derived for the TM1-weak model for the repulsive 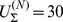 MeV potential is also included.

**Figure 19:**
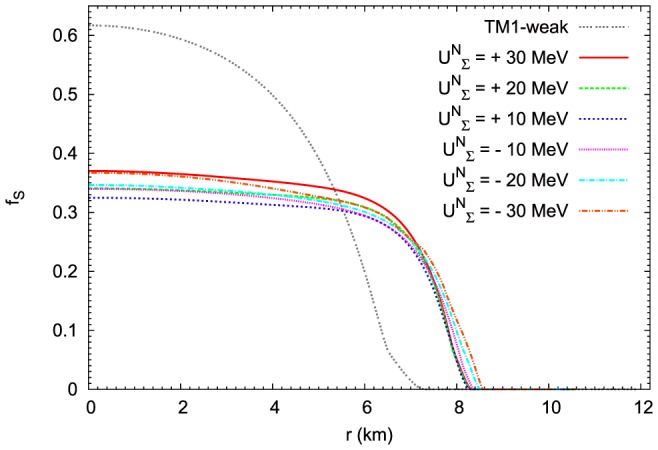
The strangeness content of the matter of the maximum mass configuration. Results obtained on the basis of the extended nonlinear model for the fixed value of the parameter 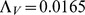. Profiles of the parameter 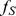 have been estimated for the 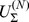 potential ranging from 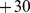 MeV to 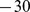 MeV. The strangeness content derived for the TM1-weak model for the repulsive 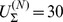 MeV potential is markedly different then those for the nonlinear model. In the case of TM1-weak model the hyperon core is more compact but with substantially enhanced hyperon content.

The results that relate to the change of asymmetry parameter 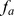 and the strangeness content of the star 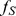 are presented in Fig. 18 and 19 respectively. In both cases the obtained results show the existence of significant differences between the TM1-weak model and the extended nonlinear model. Considering the asymmetry parameter 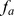 the largest differences occur in the outer part of the hyperon core where TM1-weak model leads to the enhanced asymmetry of the system. Comparing the profiles of the 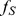 parameter it is evident that the very inner part of the hyperon core is characterized by markedly enhanced strangeness content of the matter modelled on the basis of TM1-weak parameter set in contradiction to the results obtained for the outer part of the hyperon core.

**Figure 20:**
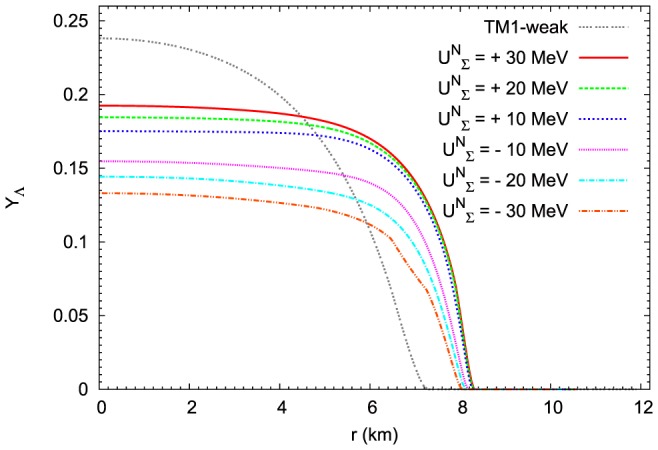
The influence of 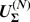 potential on the 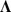 hyperon fractions for the maximum mass configuration. The figure depicts the change in the concentration of 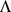 hyperons caused by different values of the 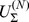 potential. 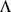 hyperons are the most abundant in the case of repulsive potential 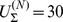 MeV. For lower value of the repulsive potential concentrations of these hyperons are reduced. The lowest concentrations of 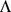 hyperons occur for the attractive potential 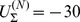 MeV. Calculations obtained for the TM1-weak model for the repulsive 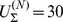 MeV potential give as a result much higher concentration of 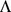 hyperon.

**Figure 21:**
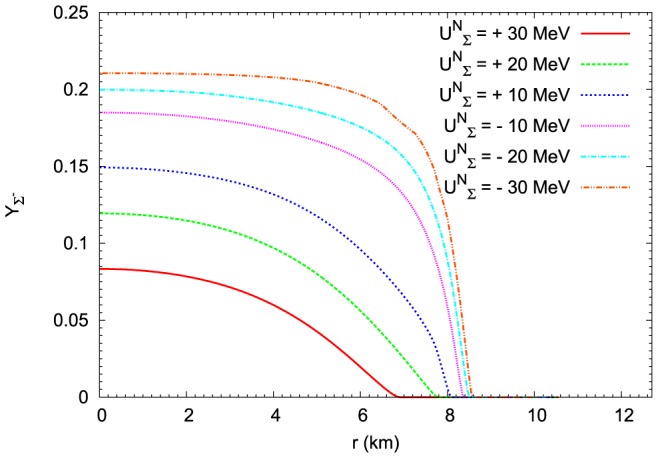
The influence of 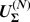 potential on the 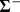 hyperon fractions for the maximum mass configuration. In the case of 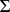 hyperons the change of the 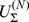 potential produces the opposite effect to that which is obtained for 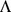 hyperon. The highest abundance of 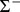 hyperons is obtained for the attractive potential 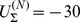 MeV. For other cases of the 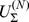 potential the concentration of 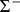 hyperons is dropping reaching the lowest value for the repulsive potential 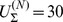 MeV. In the case of the TM1-weak model with the repulsive 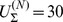 MeV potential the relative concentration of 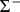 hyperons vanishes.

**Figure 22:**
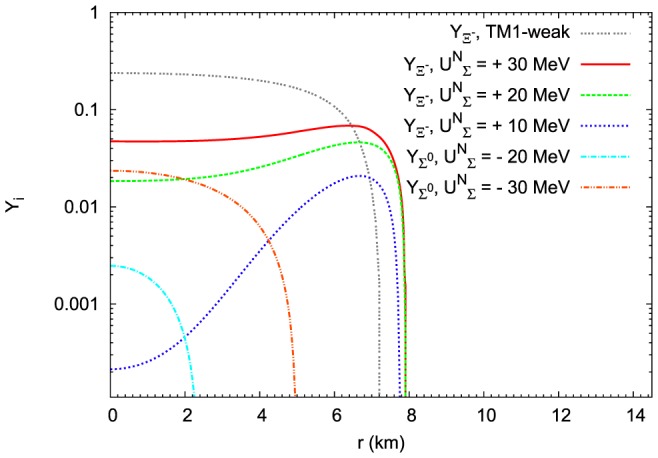
The influence of 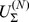 potential on the relative concentrations of 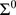 and 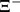 hyperons. 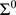
 hyperons appear inside the hyperon core for sufficiently large values of the attractive potential (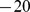 MeV and 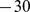 MeV). 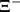 hyperons appear in the hyperon core only in the case of repulsive potential. The higher the value of the repulsive potential, the higher abundance of 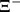 hyperons has been obtained. Calculations have been done for the maximum mass configuration.

The differences in the radial dependence of the parameters 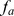 and 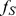 are connected with the chemical composition of the matter of neutron stars especially with the reduced abundance of hyperons in the extended nonlinear model. The influence of 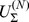 potential on the relative concentrations of 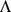 hyperons for the maximum mass configuration is depicted in Fig. 20. 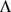 hyperons are the most abundant in the case of repulsive potential 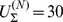 MeV. For lower value of the repulsive potential concentrations of these hyperons are reduced. The lowest concentrations of 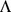 hyperons occur for the attractive potential 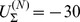 MeV. Calculations obtained for the TM1-weak model for the repulsive 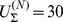 MeV potential give as a result much higher concentration of 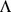 hyperon. In the case of 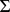 hyperons the change of the 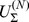 potential produces the opposite effect to that which is obtained for 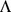 hyperon. The highest abundance of 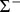 hyperons (Fig. 21) is obtained for the attractive potential 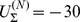 MeV. For other cases of the 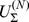 potential the concentration of 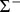 hyperons is dropping reaching the lowest value for the repulsive potential 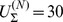 MeV. In the case of the TM1-weak model with the repulsive 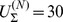 MeV potential the relative concentration of 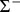 hyperons vanishes. 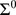 hyperons (Fig. 22) occur inside the hyperon core for sufficiently large values of the attractive potential (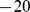 MeV and 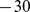 MeV). 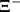 hyperons (Fig. 22) appear in the hyperon core only in the case of the repulsive potential. The higher the value of the repulsive potential, the higher the abundance of 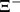 hyperons.

## Discussion

Recent observations of the binary millisecond pulsars give in the result the value of neutron star masses of the order of two solar masses. Theoretical models that describe strangeness-rich matter of a neutron star lead to much softer EoSs than those constructed for a neutron star composed of only nucleons. This significant softening of the EoS results in the low value of the maximum neutron star mass achievable in a theoretical model of a neutron star that involve hyperons and in general, leads to the inconsistency between theoretical models and observations.

One of the possible solution to this problem, is the construction of a theoretical model of the strangeness-rich matter of a neutron star that will result in the emergence of extra repulsive force in the strange sector of the system. This in turn stiffens the EoS and leads to the higher value of the maximum mass of a neutron star.

The model that was used in this paper for the description of the matter of a neutron star is based on the nonlinear realization of the chiral symmetry. The results were obtained under the assumption that nucleons do not couple to meson 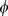. Alternative analysis of the behavior of the EoS based on 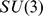 symmetry for different relativistic models have been already done and the results are included in [57]–[59]. Whereas the analysis of the EoS of hypernuclear matter within a relativistic density functional theory with density-dependent couplings has been included in [60].

An essential characteristic of the model that was used in this paper is the diverse vector meson sector which comprises different vector meson couplings. The performed analysis concerns the issue of the influence of the nonlinear 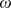, 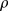 and 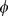 vector meson interactions on the properties of asymmetric strangeness-rich nuclear matter and consequently on neutron star parameters.

As a result, a special class of EoSs was obtained. Particular EoSs are characterised by the value of parameter 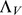, which determines the strength of the vector meson couplings. This very special form of the considered model of matter of a neutron star with hyperons gives an EoS that is much stiffer than that obtained using the standard TM1-weak model in which hyperons are introduced in a minimal fashion.

Obtained numerical solutions allow one to analyse the impact of the nonlinear vector meson couplings on the density dependence of the scalar and vector meson fields. It was shown that under the condition of the presence of nonlinear vector meson couplings the in-medium properties of baryons and mesons were changed. Especially there is a considerable modification of the effective baryon and vector meson masses.

The nonlinear vector meson couplings modify both the asymmetry and strangeness content of the system and therefore lead to a model with a reduced strangeness and an enhanced asymmetry. The stiffness of the EoS is directly related to the in-medium properties of the matter of a neutron star. It depends on value of the effective baryon and meson masses, which modify the compressibility of the matter of a neutron star and the range of interactions especially in the strange sector. The results of the analysis performed in the framework of the nonlinear model have shown that the properties of neutron stars are significantly altered by the presence of hyperons.

The analysis of the dependence of neutron star parameters on the strength of hyperon-nucleon interaction has been already done by Weissenborn et al. [56]. In this paper similar analysis was done and in this case the change of the value of hyperon-nucleon potential only slightly modifies the maximum mass of a neutron star, preferring a repulsive character of this potential. However, the changes of the hyperon-nucleon potential influence the parameters of the hyperon core of the neutron star.

The EoS for strangeness-rich matter of neutron stars calculated on the basis of the extended nonolinear model is much more stiffer then EoSs obtained for a neutron star with hyperons with the use of models in which vector meson 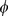 is introduced in a minimal fashion (e.g. TM1-weak model). The consequences for the parameters of neutron stars are straightforward and appear as the considerable growth of neutron star masses.

In general, the hyperon fraction is reduced in comparison to the linear models. The reduction of the hyperon population in the matter of a neutron star is related to the enhanced lepton concentrations. As an example the maximum mass configuration is presented. In this particular model the core of a neutron star reveal a hyperon inner core with the reduced 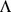 and 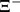 hyperon concentrations but with the enhanced population of 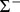 hyperons.
